# PSMA-617 inhibits proliferation and potentiates the ^177^Lu-PSMA-617-induced death of human prostate cancer cells

**DOI:** 10.1007/s00210-023-02539-w

**Published:** 2023-06-07

**Authors:** Yi Zhao, Juraj Culman, Ingolf Cascorbi, Niklas Nithack, Marlies Marx, Maaz Zuhayra, Ulf Lützen

**Affiliations:** 1 Department of Nuclear Medicine, Molecular Imaging, Diagnostics and Therapy, University Hospital Schleswig-Holstein, Campus Kiel, Kiel, Germany; 2 Institute of Experimental and Clinical Pharmacology, University Hospital Schleswig-Holstein, Campus Kiel, Kiel, Germany; 3 Central Rhine Community Hospital–Clinic for Urology and Pediatric Urology, Koblenz, Germany

**Keywords:** Prostate carcinoma cells, PSMA-617, ^177^Lu-PSMA-617, Anti-proliferation, Cell death

## Abstract

The human prostate–specific membrane antigen (PSMA) is substantially up-regulated in metastatic prostate cancer (PCa) cells. PSMA can be targeted by ^177^Lu conjugated to PSMA-617, a high-affinity ligand for the PSMA. The binding of the radioligand, ^177^Lu-PSMA-617, results in its internalisation and delivery of β-radiation into the cancer cells. However, PSMA-617, a component of the final product in the synthesis of the radioligand, may also play a role in the pathophysiology of PCa cells. The present study aimed to clarify the effects of PSMA-617 (10, 50 and 100 nM) on the expression of PSMA in PSMA-positive LNCaP cells, their proliferation, ^177^Lu-PSMA-617-induced cell death by WST-1 and lactate dehydrogenase assays, immunohistochemistry, western blotting, immunofluorescence staining and uptake of ^177^Lu-PSMA-617. PSMA-617 at 100 nM concentration induced cell-growth arrest, down-regulated cyclin D1 and cyclin E1 (by 43 and 36%, respectively) and up-regulated the cyclin-dependent kinase inhibitor p21^Waf1/Cip1^ (by 48%). Immunofluorescence staining demonstrated reduced content of DNA, pointing to a lower rate of cell division. PSMA-617 (up to 100 nM) did not alter the uptake of ^177^Lu-PSMA-617 into the LNCaP cells. Interestingly, simultaneous treatment with ^177^Lu-PSMA-617 and PSMA-617 for 24 and 48 h substantially potentiated the cell-death promoting effects of the radioligand. In conclusion, the combination of impeding tumour cell proliferation by PSMA-617 and its potentiation of the radiation-induced cell death brought about by ^177^Lu-PSMA-617 in PCa cells may considerably improve the outcome of the radiation therapy with ^177^Lu-PSMA-617, especially in patients with decreased radiosensitivity of PCa cells to the radioligand.

## Introduction

Radioligand therapy is a new, attractive therapeutic option for the treatment of metastatic, castration-resistant prostate cancer (mPCa). The therapeutic approach is based on the interaction of small, high affinity ligands with the prostate-specific membrane antigen (PSMA). PSMA is a carboxypeptidase, also known as folate hydrolase I or glutamate carboxypeptidase II. PSMA is excessively, 100–1000 times up-regulated in advanced-stage PCa, in fact PSMA expression correlates with increased growth and invasiveness of the tumour (Perner et al. 2007; El Fakiri et al. 2021). Although the exact role of PSMA in the progression of PCa has not yet been fully elucidated, few experimental data indicates that up-regulated PSMA belongs to the factors directly mediating tumour growth and aggressiveness (Perico et al. 2016; O'Keefe et al. 2018). For instance, PSMA knock-down inhibited the proliferation and migration of LNCaP cells and reduced the expression of the androgen receptor, PSA and transcription factors c-Fos and FosB (Hong et al. 2022). Due to the close relation to tumour progression, PSMA emerged as an attractive target for a more efficient diagnosis and therapy. The first urea-based PSMA inhibitors based on Glu-urea-Lys binding motif developed by Kozikowski and co-workers (et al. 2001) showed effective tumour targeting but less optimal pharmacokinetic properties. The modifications to the molecule between the binding motif and the radiometal chelator DOTA increased the affinity and tumour retention as well as pharmacokinetic properties (Benešová et al. 2016). PSMA-617 (vipivotide tetraxetan), a high-affinity PSMA inhibitor (Ki 0.37 nM), conjugated with ^177^Lu was the most promising radioligand interacting with PSMA (Benešová et al. 2015). After binding of ^177^Lu-PSMA-617 to PSMA, the complex is internalised and transported into PCa cells, where ^177^Lu releases high-energy beta-particles which preferentially destroy cancer cells. The first clinical study and a retrospective multicentre analysis have demonstrated that ^177^Lu-PSMA-617 is a promising therapeutic option for therapy of mPCa (Ahmadzadehfar et al. 2015; Kratochwil et al. 2016; Rahbar et al. 2017). Subsequent clinical trials demonstrated a substantial anti-tumour activity, favourable safety and few side effects as reviewed by Hennrich and Eder 2022, and Ramnaraign and Sartor 2023. The VISION study, a prospective open-label phase III trial, demonstrated that ^177^Lu-PSMA-617 in combination with standard therapy significantly reduced the overall mortality and disease progression in patients with advanced mPCa (Sartor et al. 2021). Based on the results of the VISION trial, ^177^Lu-vipivotide tetraxetan (Pluvicto®) was approved by the FDA and EMA in 2022 for the treatment of PSMA-positive metastatic castration-resistant PCa. A number of further PSMA-targeted radiopharmaceuticals are under development (Sartor and Baghian 2022; Jang et al. 2023).

A few studies report on the effects of PSMA-targeting ligands on proliferation of healthy and tumour cells. Inhibition of cell proliferation in vitro and in vivo was observed in fibroblasts expressing the fibroblast activation protein and in PSMA-positive neovasculature tissue of the tumour microenvironment after their exposure to [^177^Lu] Lu-iFAP/iPSMA nanoparticles (Hernández-Jiménez et al. 2022). Enhanced uptake of micelle-encapsulating wogonin (2-(3-((S)-5-amino-1 carboxypentyl) ureido) pentanedioic acid-modified polyethylene glycol-cholesterol micelles containing wogonin) into PSMA-positive PCa cells reduced their proliferation and exerted cytotoxic effects by activation of the intrinsic or extrinsic apoptosis pathways (Zhang et al. 2016). Finally, conjugated PSMA-docetaxel showed the cytotoxic potential to inhibit tumour growth of PCa close to unmodified docetaxel, an effect accompanied with lower toxicity than docetaxel itself (Machulkin et al. 2022).

The pathophysiological processes occurring in PCa cells exposed to PSMA-617 have not yet been comprehensively studied, although PSMA-617 is, in general, a component of the final product of the ^177^Lu-PSMA-617 synthesis. We have therefore investigated the effects of PSMA-617 in PSMA-positive PCa cells, (LNCaP cells, lymph node carcinoma of the prostate) on binding of ^177^Lu-PSMA-617 to PSMA, tumour growth, cell-cycle arrest, cell death and the corresponding underlying mechanisms. We report that PSMA-617 inhibits proliferation and induces cell cycle arrest in the G1 stage in LNCaP cells and considerably potentiates the cytotoxic effect of ^177^Lu-PSMA-617.

## Materials and methods

### Chemicals and reagents

#### Antibodies (Ab)

Rabbit monoclonal anti cyclin D1, mouse monoclonal anti Cyclin E1, mouse monoclonal anti p21^Waf1/Cip1^ (p21) and rabbit monoclonal anti p27^Kip1^ (p27) (Cell Signalling Technology); mouse monoclonal anti PSMA (Abcam); mouse monoclonal anti β-actin (Sigma-Aldrich); horseradish peroxidase-conjugated secondary anti-rabbit or anti-mouse IgGs (Amersham); Fluor® 488-conjugated donkey anti-mouse IgG (Molecular Probes).

#### Chemicals and kits

PSMA-617 (ABX Advanced Biochemical Compounds); WST-1 assay kit and Cytotoxicity detection kit (lactate dehydrogenase, LDH) (Roche); Propidium Iodide(PI) Reagent (Invitrogen); cresyl violet (Sigma-Aldrich); PRMI 1640 culture medium, HAM’-12 medium, foetal bovine serum (FBS) and penicillin/streptomycin solution (10,000 U/ml) (Gibco™); Cellytic™ MT Cell Lysis Reagent (Sigma-Aldrich); halt protease and phosphatase inhibitor and Pierce™ BCA protein assay kit (Thermo Scientific); Western Blotting Luminol Reagent (Santa Cruz Biotechnology); Immobilon-P polyvinylidene difluoride membrane (PVDF) (Millipore); high performance chemiluminescence film (Amersham International plc); Western Blot Stripping Buffer (Thermo Scientific). All other chemical substances, which are not explicitly mentioned, were purchased from Sigma-Aldrich or Merck.

### Cell cultures

LNCaP cells (ACC 256), a human prostate carcinoma cell line, were purchased from DSMZ (German Collection of Microorganisms and Cell Culture GmbH). The cells were cultured in RPMI 1640 medium containing 10% heat inactivated FBS, 100 U/ml penicillin and 100 µg/ml streptomycin. PC3 cells (ATCC, CRL-1435), kindly provided by Dr. Tiwari from Molecular Imaging North Competence Centre, University of Kiel, Germany, were grown in the culture medium consisting of RPMI 1640 and HAM′-12, 1:1, supplemented with 10% heat inactivated FBS, 100 U/ml penicillin and 100 µg/ml streptomycin. All cells grew as a monolayer in dishes at 37 °C in a humidified atmosphere of air/CO_2_ (19:1). For cell passages, trypsin/EDTA (0.025% trypsin and 0.02% EDTA) was used.

### WST-1 assay

Cell proliferation was assessed by the WST-1 reagent according to the manufactures’ instructions. Cells were grown in 24-well culture plates and exposed to PSMA-617 (10, 50 or 100 nM) for 24 or 48 h, respectively. Aliquots of the culture medium from each well were taken for LDH assay (see below). The cells were then incubated with 10% WST-1 reagent for 4 h. One hundred microliter of supernatant were transferred into a 96-well plate and measured with Multi-Well Spectrophotometer (Elisa Reader, TECAN Infinite 200) at 460 nm wavelength. The absorbance at 600 nm served as a reference. The absorbance directly correlates with the number of viable cells. Each sample was measured in triplicate.

### Lactate dehydrogenase (LDH) assay

The assessment of the cell membrane integrity and cytotoxicity based on the measurement of LDH activity released from damaged cell into the culture medium were determined by the Cytotoxicity detection kit according to the manufacturers’ recommendations and as published previously (Zhao et al. 2015). For each sample, 10 µl of the culture medium and 90 µl PBS (phosphate buffered saline) were added to 100 µl of working solution in 96-well plates and incubated for 30 min at RT in dark. Afterwards, the absorbance (450 nm) of all samples and the controls were detected with a microplate reader. The absorbance at 600 nm served as a reference. Each sample was measured in triplicate.

### Protein isolation and western blot analysis

Cells were lysed in Cellytic™ MT Cell Lysis Reagent containing 1% of the halt protease and phosphatase inhibitor on ice for 30 min. After short incubation (5 min at 95 °C), the lysates were sonicated and centrifuged (15,000 × g at 4 °C for 15 min). The protein concentration in the supernatant was measured by the Pierce™ BCA Protein assay kit according to the manufacturer’s instruction. Extracts equivalent to 15 or 30 µg of total proteins per lane were loaded and separated on 10% or 12% SDS–polyacrylamide gels and transferred to PVDF membranes. The membranes were blocked and incubated overnight with primary Ab against PSMA (1:5000), cyclin D1 (1:1000), cyclin E1 (1:3000), p21 (1:2000) or p27 (1:1000). On the next day, the membranes were washed and incubated with the corresponding horseradish peroxidase-conjugated secondary Ab. Western blots were developed with Western Blotting Luminol Reagent on high performance chemiluminescence film. For the re-staining, the blots were stripped in the Restore Western Bolt Stripping Buffer, washed in Tween-Tris-Buffered saline, with 1% Tween and blotted again. To normalise the protein content of each lane, all membranes were blotted with anti-β-actin Ab (1:10,000). The films were scanned and quantified using the quantification software (Quantity One, Bio-Rad).

The determination of PSMA in LNCaP or PC3 cells was carried out in cells cultured in the appropriate culture medium till 80–90% confluence was reached. The effect of PSMA-617 on the expression of the cell cycle regulators was quantified in LNCaP cells exposed to vehicle (controls) or PSMA-617 (10, 50 or 100 nM) at 37 °C for 24 or 48 h, respectively.

### Immunofluorescence staining for the PSMA and DNA

Cells grown on cover slips (Becton Dickinson) in 4-well plates were exposed to vehicle or PSMA-617 (100 nM) for 24 h. The cells were fixed with 4% paraformaldehyde (PFA) for 30 min at room temperature (RT), washed twice with 2 × SSC buffer (0.3 M NaCl, 0.03 M sodium citrate, pH 7.0), treated with 100 µg/ml DNase-free RNase diluted in 2 × SSC buffer and incubated at 37 °C for 20 min to destroy RNA. The cells were washed three times with PBS (phosphate buffered saline) containing 0.02% of Triton (PBST), permeabilised with 0.2% saponin in 0.1% BSA (bovine serum albumin) for 30 min at RT, incubated in the block solution containing 1% BSA for 30 min at RT and exposed to the primary Ab, mouse anti-human PSMA (1:3000), at 4 °C overnight. Following three washing steps with PBST, the cells were incubated with Alexa Fluor®488-conjugated donkey anti-mouse 2^nd^ Ab (1:500) in 0.5% BSA for 1 h at RT in dark. After the incubation and washing with PBST and twice with 2 × SSC buffer, PI regent (500 nM in 2 × SSC buffer) was added (300 µl/well) and the cells were incubated for 2 min at RT. The cells were washed three times with 2 × SSC buffer, dried in the dark and mounted in Prolong® Gold Antifade Reagent. Immunofluorescence analyses were carried out by a microscope (Leica DMR Microscope system equipped with CC 12 digital camera).

### Cresyl violet staining

LNCaP cells cultured in 24-well plates were exposed to vehicle or to 10, 50 and 100 nM PSMA-617 for 24 or 48 h, respectively. The cells were washed three times with PBS and fixed with 4% PFA/PBS at RT for 1 h. After washing three times with PBS and twice with distilled water, the cells were incubated with 0.1% cresyl violet solution for 20 min at RT. Cresyl violet was washed out with distilled water, the stained cells were dried and photographed under an inverted light microscope (OLYMPUS IX73 Microscope system equipped with OLYMPUS DP74 digital camera).

### Synthesis and quality control of ^177^Lu-PSMA-617

No-carrier added ^177^LuCl_3_ solution (⁓36–44 Gbq/ml) (EndolucinBeta®) was purchased from ITM (ITM Isotopen Technologien). PSMA-617 (60 µg) mixed with 740 µl of gentensic solution (3 mg/ml dissolved in 0.4 M sodium acetate, pH = 4.6) was added to 163 µl of the ^177^LuCl_3_ solution. The buffered solution was heated for 20 min at 92 °C and diluted with saline to reach a final activity of 1 GBq/ml. Quality control was performed using the TLC scanner and HPLC according to the Guideline Monographs on Radiopharmaceutical Preparations (Gillings et al. 2020) spotting 0.3 µl aliquots on the Chromatography strips (Biodex) employing 0.1 M citric acid buffer (pH 5.5) as eluent or on the ITLC-SG plates (Varian) using 1 M NaAc/MeOH (1:1) as eluent. The strips and plates were scanned using a flat-bed scanner (Rita Star, Raytest-Isotopenmessgeräte GmbH). The radiochemical and chemical purity were determined by a gradient HPLC method. The analytical HPLC apparatus was equipped with a radiation detector and an UV (220 nm) detector. HPLC separations were performed on Chromolith columns (3 × 100 mm). The gradient eluent consisted of mobile phase A (water containing 0.1% TFA) and mobile phase B (acetonitrile containing 0.1% TFA). Flow rate was 1.0 ml/min. Starting with 100%A/0%B, the gradient was increased to 100% B over 8 min. The retention time of free ^177^LuCl_3_ was 0.8 min and that of ^177^Lu-PSMA-617 4.7 min, respectively.

### Effect of PSMA-617 on the uptake of ^177^Lu-PSMA-617 into LNCaP cells

LNCaP cells (2 × 10^6^) were plated onto 10-cm Petri dishes and incubated for 2–3 days. The cells were simultaneously treated with ^177^Lu-PSMA-617 (0.44 nM, 2 MBq/4 ml/dish) and PSMA-617 (10, 50 or 100 nM) for 30 min, rinsed twice with 4 ml of ice-cold saline, scraped using 1-ml cold saline, transferred to an Eppendorf-tube and centrifuged (2000 rpm) for 5 min at 4 °C. The optimal concentration of ^177^Lu-PSMA-617 was established in preliminary experiments. The radioactivity of the cell pellets was measured with a γ-counter (ISOMED 200). The protein content of the cell pellets was measured as described above. The counts of individual samples were normalised for protein content. The data is expressed as cpm/µg of protein.

### Effects of PSMA-617 on the anti-cancer efficacy of ^177^Lu-PSMA-617 in LNCaP cells

LNCaP cells, cultured in a 24-well plate (approximately 1 × 10^5^ cells/well), were incubated with ^177^Lu-PSMA-617 (0.18 nM, 100 KBq/0.5 ml/well) and vehicle (controls) or with 50 or 100 nM PSMA-617 for 24 (*n* = 12) or 48 h (*n* = 8), respectively. The concentration of ^177^Lu-PSMA-617 used in this experimental setting (0.18 nM) was 2.5–times lower than that (0.44 nM) employed in the experiments examining the uptake of ^177^Lu-PSMA-617 into LNCaP cells. ^177^Lu-PSMA-617 concentration of 0.44 nM would promote cell death within few hours. Aliquots of the culture medium were used for LDH-assay. Each sample was measured in triplicate.

### Statistical analyses

The results are expressed as the mean ± SD of triplicate determinations from 3–5 separate experiments. The distribution of the sampled data was analysed by Kolmogorov–Smirnov’s test and the homogeneity of variance was tested by Bartlett’s test. The statistical evaluation of the data was carried out by one-way analysis of variance (ANOVA) followed by a post hoc Bonferroni test for pairwise comparisons or by the Kruskal–Wallis test followed by a post hoc Dunn’s test, using the statistics software GraphPad InStat 3.1. Statistical significance was accepted at *P* < 0.05.

## Results

### Expression of PSMA and affinity test in PC3 and LNCaP cells

**Fig. 1:**
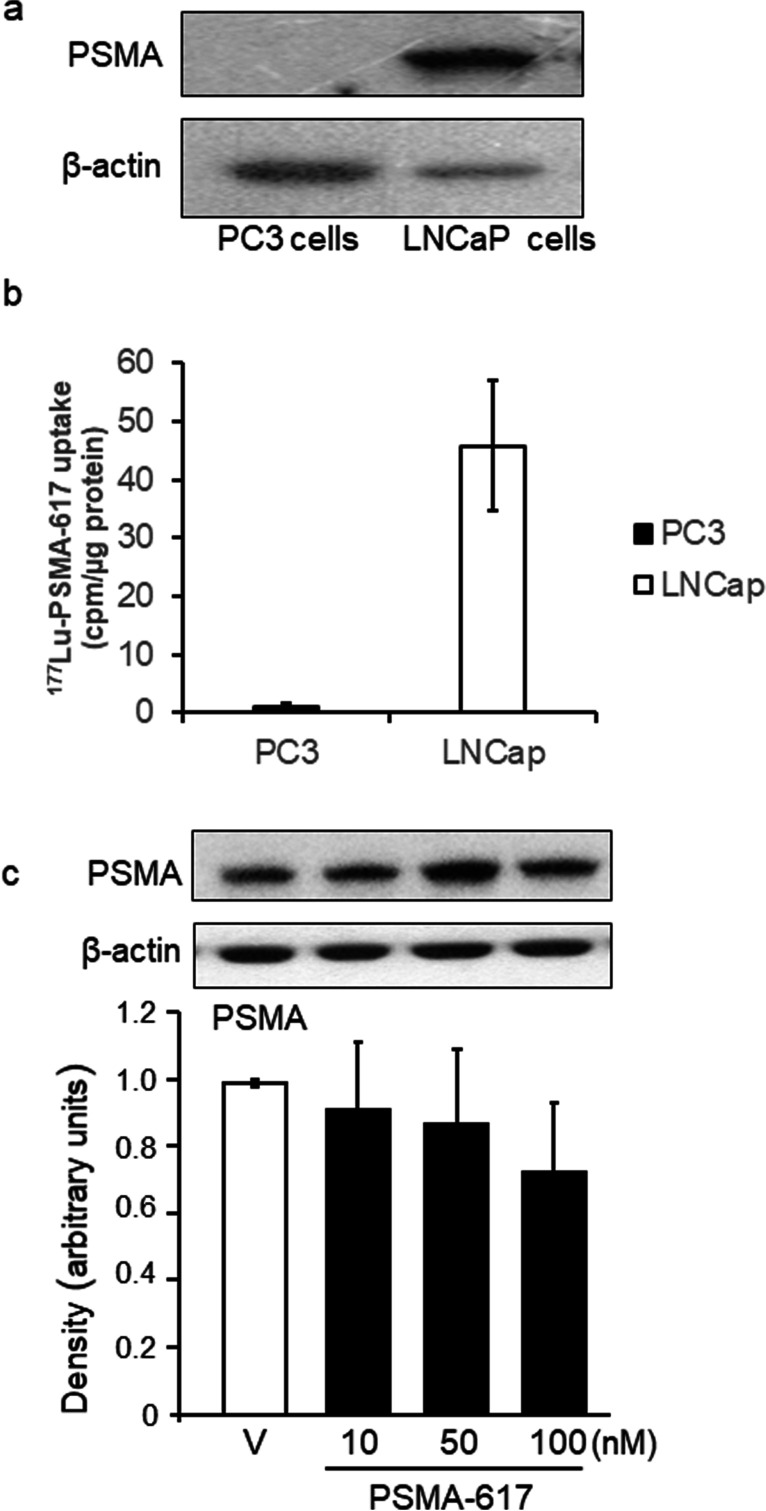
**a** PSMA is highly expressed in LNCaP cells, but not in PC3 cells (*n* = 3). **b** LNCaP cells intensively accumulate ^177^Lu-PSMA-617, no uptake of the radioligand was detected in PC3 cells (*n* = 9). Data is expressed as the means ± SD. **c** Western blot analysis of PSMA-617 in LNCaP cell treated with vehicle (V) (empty column) or 10, 50 and 100 nM PSMA-617 (solid columns) for 24 h. Representative blots and graphical analysis are shown. Data is expressed as the means ± SD. PSMA-617 does not alter PSMA expression in LNCaP cells (*n* = 5) (one-way ANOVA)

The expression of PSMA in the LNCaP and PC3 cell lines was analysed in the protein fraction using western blotting. As expected, PC3 cells did not express PSMA; however, high concentration of PSMA was detected in LNCaP cells (Fig. 1a). Accordingly, ^177^Lu-PSMA-617 displayed high affinity uptake into LNCaP cells, but not into PC3 cells (Fig. 1b).

### Effect of PSMA-617 on PSMA expression

Western blot analysis demonstrated that increasing concentrations (10, 50 and 100 nM) of PSMA-617 did not significantly alter the PSMA protein levels in LNCaP cells after 24 h of treatment, (*n* = 5) (one-way ANOVA) (Fig. 1c).

### PSMA-617 and cell death

**Fig. 2:**
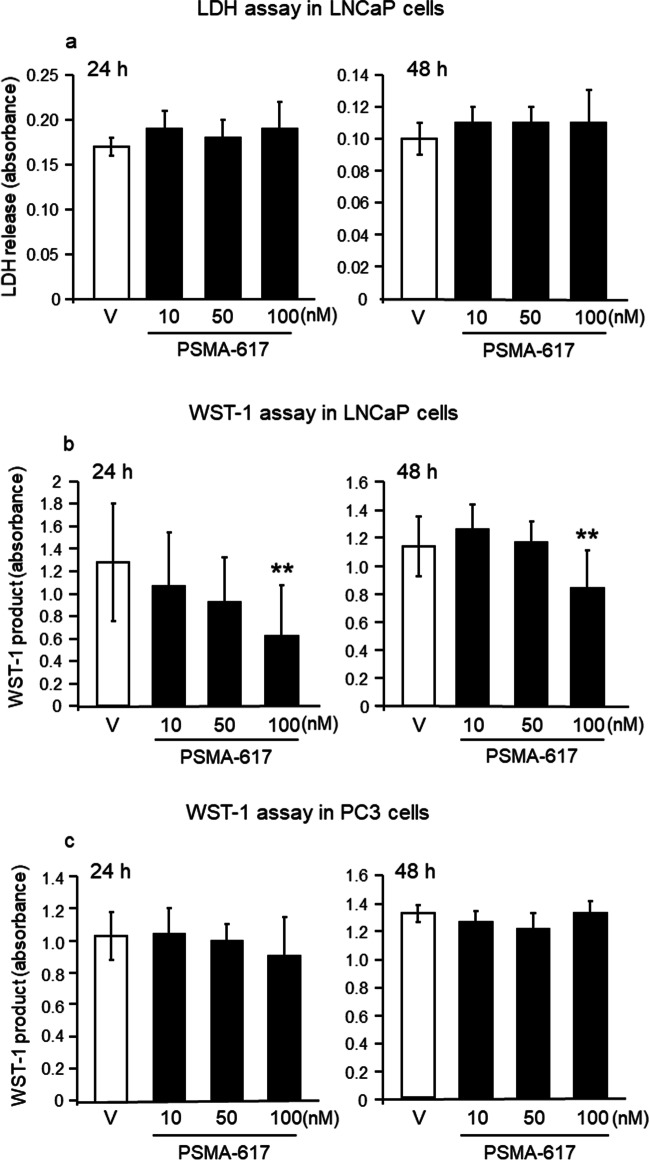
Effect of PSMA-617 on cell death (upper panels) and proliferation of LNCaP and PC3 cells (lower panels). Empty columns, cells treated with vehicle (V); solid columns, cells treated with PSMA-617. Exposure of LNCaP cells to PSMA-617 for 24 (*n* = 12) or 48 h (*n* = 10) did not induce cell death (**a**), but significantly reduced their proliferation rates (*n* = 12 each group) (**b**). No proliferative effects were detected in PC3 cells (*n* = 10) (**c**). Results are expressed as the means ± SD. Statistical comparison with vehicle-treated cells: ^**^P < 0.01, calculated by Kruskall-Wallis test (24 h) or by one-way ANOVA (48 h) followed by a post-hoc Dunn’s test and Bonferroni test, respectively

As surrogate parameter of cytotoxicity, LDH release into the culture medium was quantified in LNCaP cells incubated with increasing concentrations of PSMA-617 (10, 50, 100 nM). Compared to vehicle-treated cells, PSMA-617 did not increase LDH release from the cells at any used dose, indicating that even high doses of PSMA-617 did not induce cell death (24 h: *n* = 12, 48 h: *n* = 10) (Fig. 2a).

### PSMA-617 and cell proliferation

WST-1 analysis was carried out to detect the relative proliferation rates of LNCaP and PC3 cells after their incubation with PSMA-617 for 24 (*n* = 12) or 48 h (*n* = 12). PSMA-617 (100 nM) significantly reduced proliferation of LNCaP cells after 24 h by 50% (*P* < 0.01) and 48 h by 26% (*P* < 0.01) (Fig. 2b). This effect was detected only in LNCaP cells but not in PC3 cells (Fig. 2c), suggesting that the growth-inhibitory effect of PSMA-617 clearly depends on the PSMA expression.

**Fig. 3:**
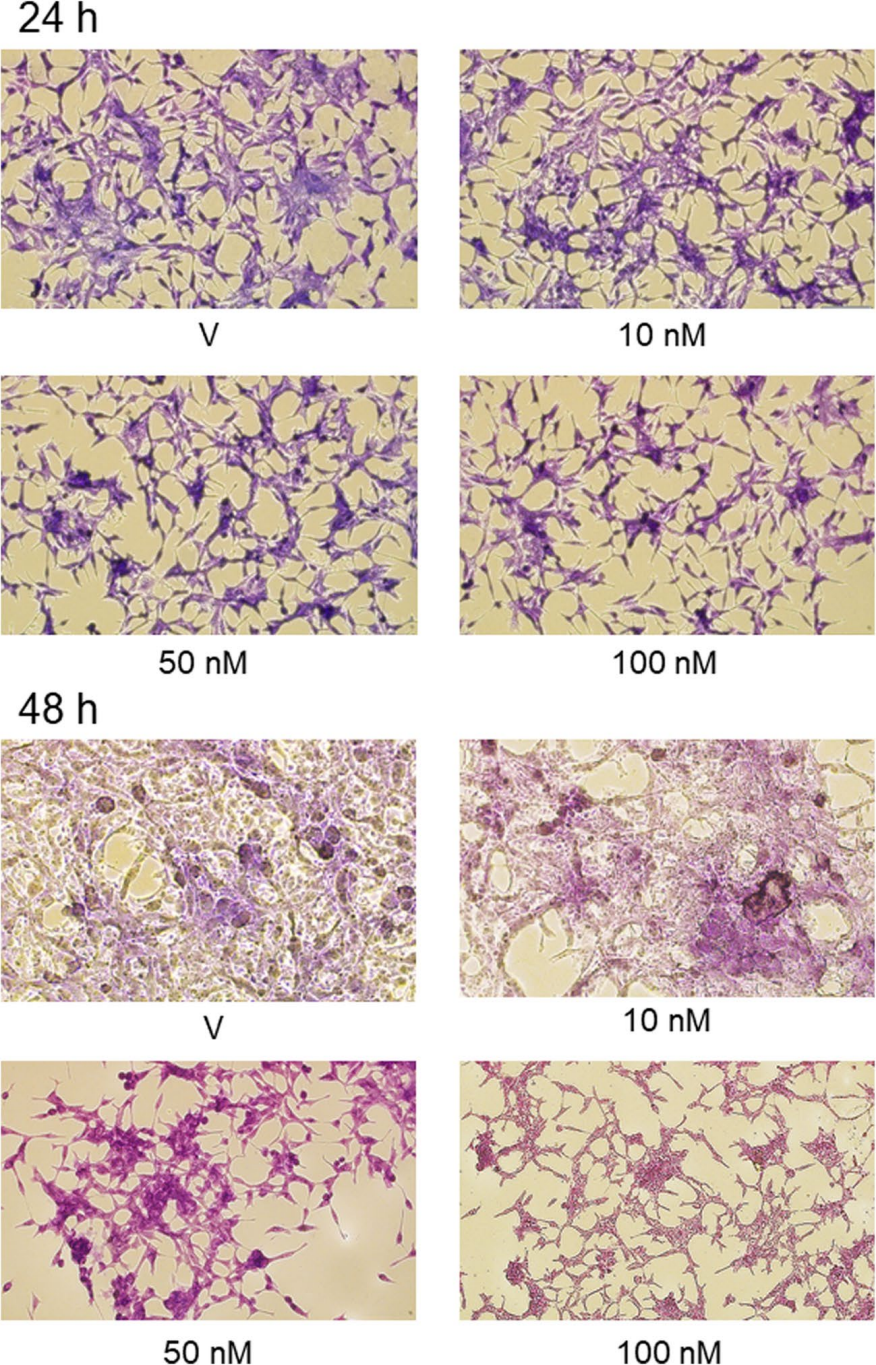
Effects of incubation of LNCaP cell with PSMA-617 for 24 and 48 h on their morphology. LNCaP cells treated with vehicle rapidly proliferate, display irregular size and shape and nuclear atypia, especially after 48 h-lasting incubation. PSMA-617 decreased cell proliferation and, interestingly, the cells acquired an elongated morphology and formed processes (40 × magnification)

Additional experiments employing cresyl violet staining confirmed the anti-proliferative effects of PSMA-617 in LNCaP cells. Cells incubated in the absence of PSMA-617 (control group) showed an invasive growth, conglomerates of cell clusters, especially pronounced after incubation for 48 h. The cell numbers were lower in cells treated with PSMA-617 and the cells acquired an elongated morphology (Fig. 3).

### Mechanism of the anti-proliferative effects of PSMA-617 in LNCaP cells

**Fig. 4:**
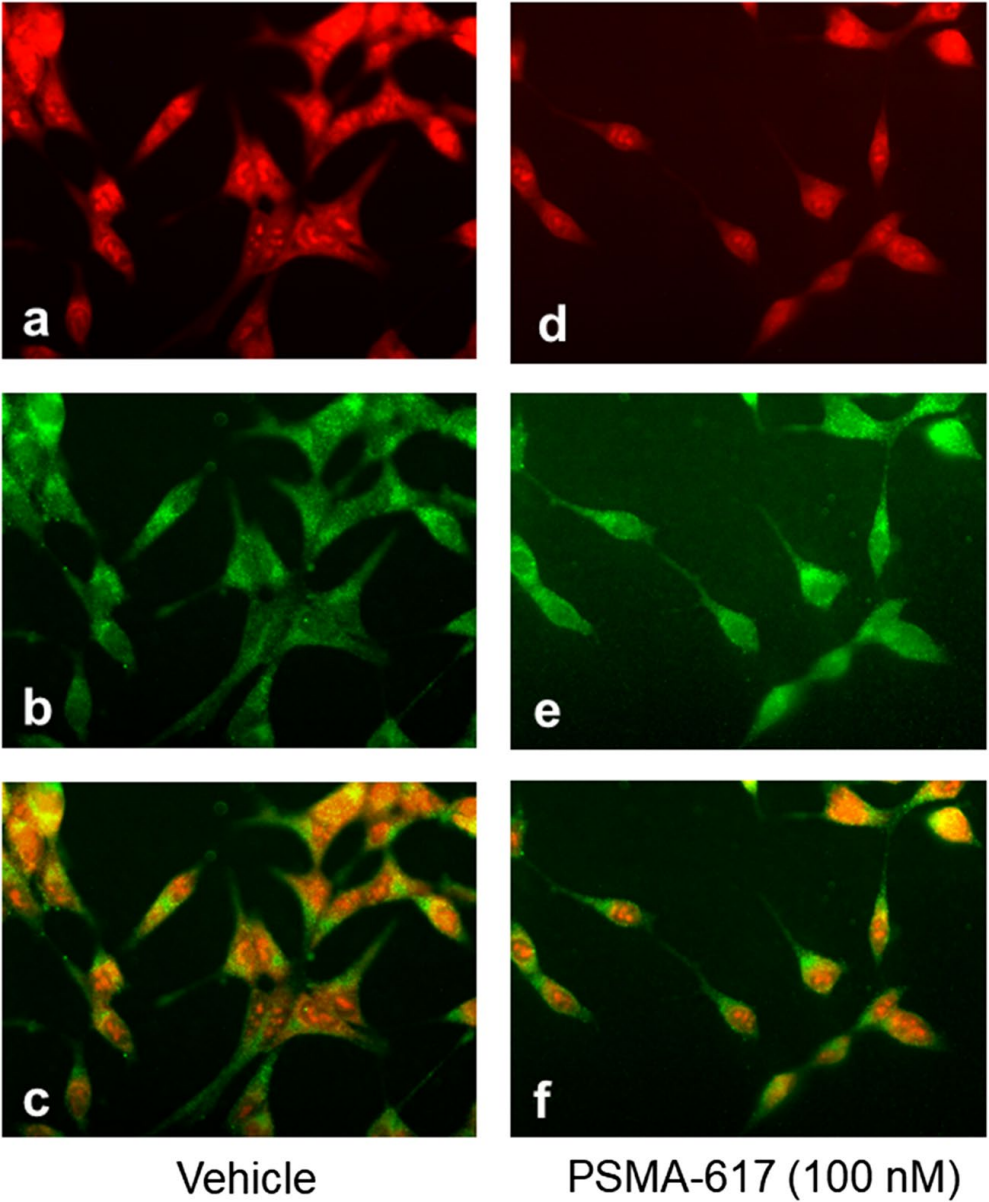
Immunofluorescence staining for DNA (red, **a**, **d**) and PSMA (green, **b**, **e**) in LNCaP cells incubated with vehicle (left panels) or with 100 nM PSMA-617 (right panels) for 24 h. Vehicle-treated cells showed greater amounts of DNA (intense red staining), irregular form of nuclei (**a**). PSMA-617 did not alter the intensity of staining for PSMA (**b**, **e**). No overlapped immunoreactivity for DNA (localised in the nuclei) and PSMA (localised in the cytoplasm or cell membrane) was observed (**c**, **f**)

Tumour cells, which rapidly divide, contain high amounts of DNA. Labelling DNA with propidium iodide allows for fluorescence-based analysis of the cell cycle, as the amount of DNA doubles between G1 and G2 phases. Figure 4 shows double immunofluorescence staining for DNA (red) and PSMA (green) in LNCaP cells exposed to vehicle (controls) or PSMA-617 (100 nM) for 24 h. An intensive red fluorescent staining, irregular size and shape of the nuclei and multinucleate cells were observed in cells treated with vehicle (Fig. 4a–c). Treatment of LNCaP cells with PSMA-617 (100 nM) reduced qualitatively the DNA content, pointing to a lower rate of cell division (Fig. 4d–f).

**Fig. 5:**
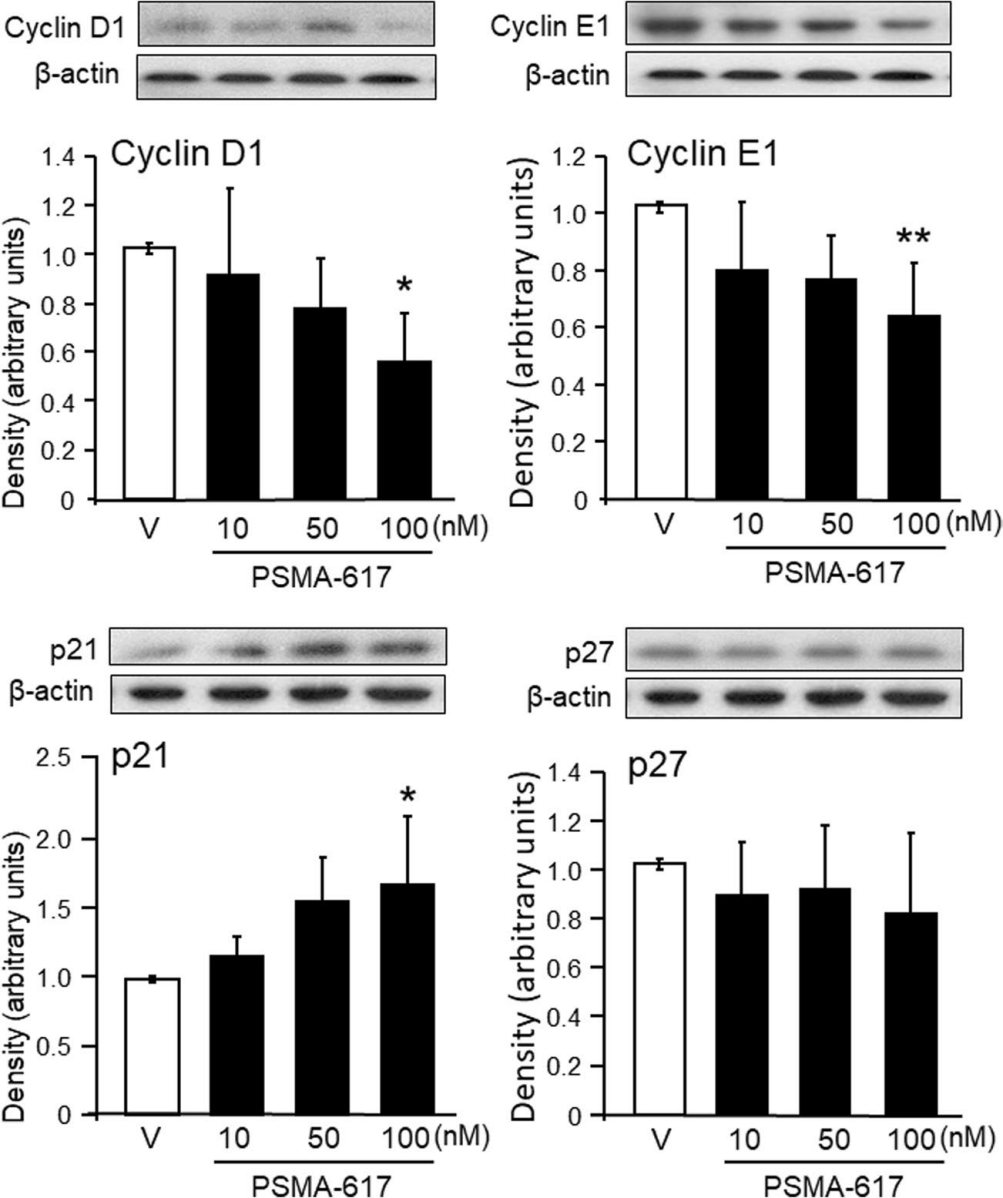
Western blot analysis of cyclin D1 (*n* = 5) and E1 (*n* = 5) (upper panels) and p21 (*n* = 5) and p27 (*n* = 9) (lower panels) in LNCaP cells treated with vehicle (controls) (empty columns) or exposed to various concentration of PSMA-617 (solid columns). Representative blots and graphical analysis are shown. Results are expressed as the means ± SD. Statistical comparison with controls: ^*^P <0.05 and ^**^P < 0.01, calculated by one-way ANOVA followed by a post hoc Bonferroni test

To further explore the effect of PSMA-617 on the cell cycle regulation, western blotting was applied to quantify the protein levels of cyclin D1 and cyclin E1 in LNCaP cells incubated for 24 h with different concentrations of PSMA-617. PSMA-617 gradually decreased cyclin D1 (*n* = 5) and cyclin E1 (*n* = 5), a significant reduction (by 43 and 36%, respectively) was detected at a concentration of 100 nM (Fig. 5, upper panels).

The quantification of cyclin-dependent kinase (CDK) inhibitors p21 (*n* = 5) and p27 (*n* = 9) levels in LNCaP cells treated with different concentration of PSMA-617 revealed that p21 but not p27 was dose-dependently up-regulated (by 48%) (Fig. 5, lower panels).

### Effect of PSMA-617 on uptake of ^177^Lu-PSMA-617 into LNCaP cells

**Fig. 6:**
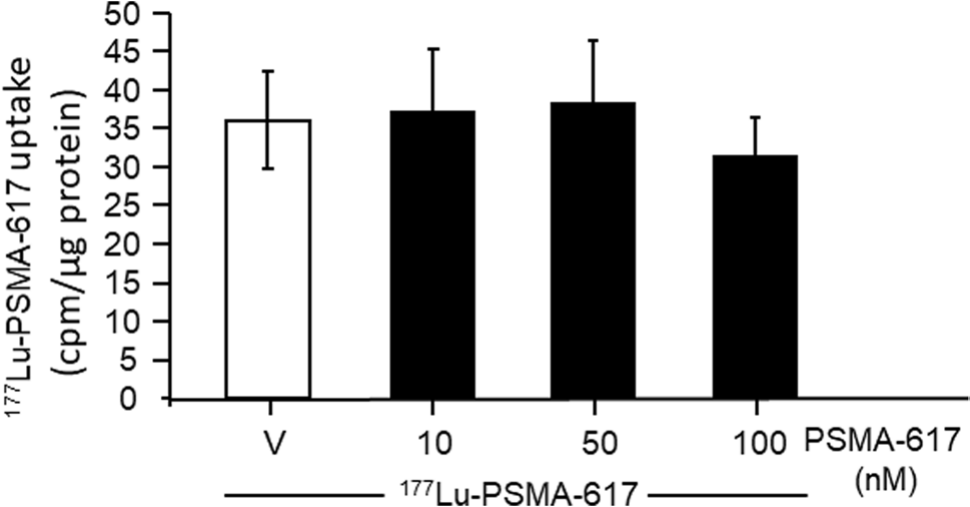
Effects of PSMA-617 (10, 50 and 100 nM) (solid columns) on the uptake of ^177^Lu-PSMA-617 into LNCaP cells. Empty column, cells treated with vehicle. Results are expressed as the means ± SD. No significant differences among the groups were found (one-way ANOVA)

Both ^177^Lu-PSMA-617 and its non-radioactive precursor, PSMA-617, bind to the PSMA on the cell membrane. Therefore, PSMA-617 could compete with the ^177^Lu-PSMA-617- binding to LNCaP cells and, consequently may reduce its cytotoxic effects. However, Fig. 6 shows that PSMA-617 concentrations up to 100 nM did not impair the ^177^Lu-PSMA-617 uptake into the LNCaP cells (*n* = 9).

### Effect of PSMA-617 on anti-cancer efficacy of ^177^Lu-PSMA-617 in LNCaP cells

**Fig. 7:**
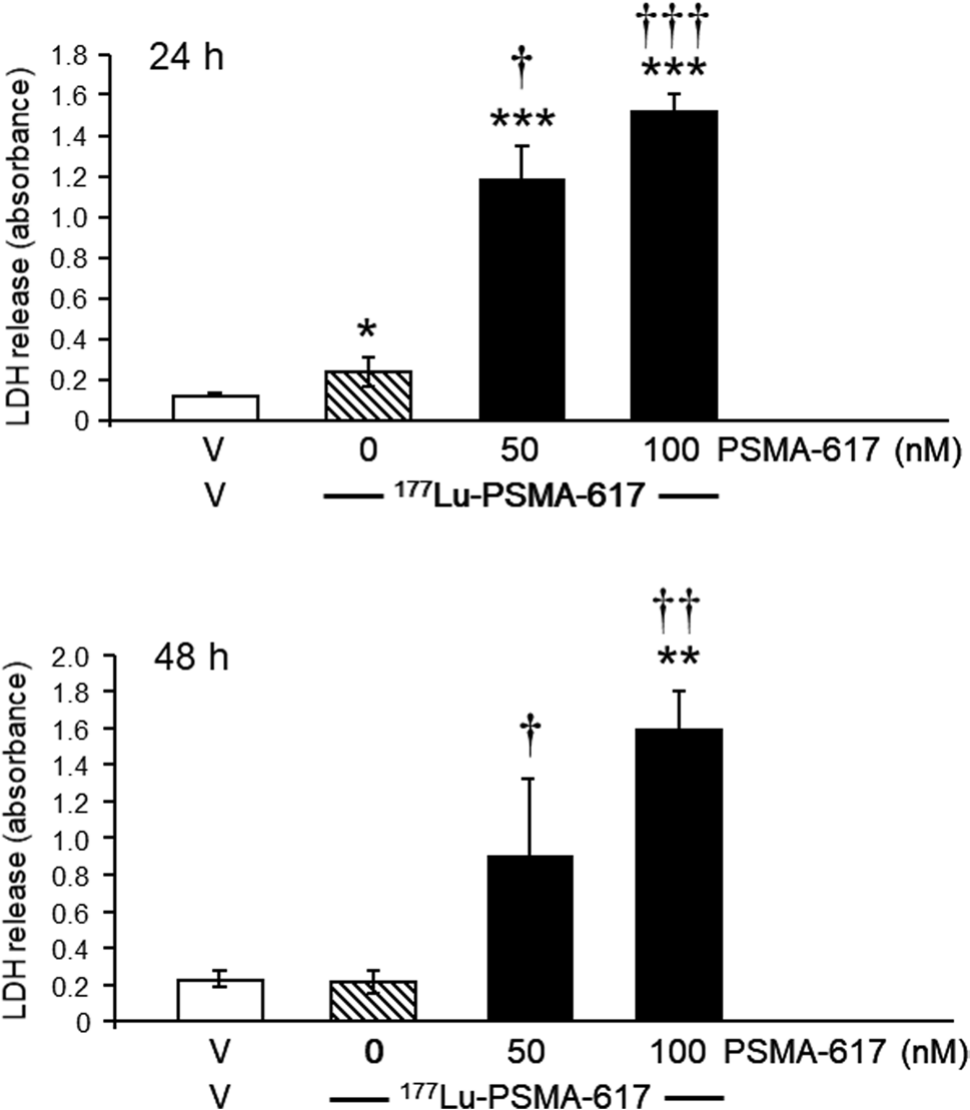
Assessment of cell death in LNCaP cell incubated with vehicle (V) (empty columns) or with ^177^Lu-PSMA-617 in the presence (solid columns) or absence of PSMA-617 (hatched columns) for 24 (*n* = 12) or 48 h (*n* = 10). PSMA-617 potentiated the cell death-promoting effects of ^177^Lu-PSMA-617. Results are expressed as the means ± SD. Statistical comparisons with the vehicle-treated group: ^*^P < 0.05, ^**^P < 0.01 and ^***^P < 0.001, and with the group exposed to ^177^Lu-PSMA-617: ^†^*P* < 0.05, ^††^*P* < 0.01 and ^†††^*P* < 0.001, calculated by Kruskal–Wallis test followed by a post hoc Dunn’s test

To investigate whether the precursor PSMA-617 interferes with cell death induced by ^177^Lu-PSMA-617, LNCaP cells were treated with ^177^Lu-PSMA-617 (100 KBq/well) combined with different concentrations of PSMA-617. PSMA-617 at 50 nM and 100 nM concentrations significantly potentiated the ^177^Lu-PSMA-617-induced LDH release from LNCaP cells after a 24 h incubation (each group *n* = 12) (Fig. 7). Although ^177^Lu-PSMA-617 alone failed to induce cell death 48 h after incubation, strong cytotoxic effects were observed in cells incubated with both ligands in parallel (each group *n* = 8) (Fig. 7).

## Discussion

The PSMA-targeted radioligand, ^177^Lu-PSMA-617, has been clinically tested in the therapy of PCa since 2015 (Hennrich and Eder 2022) and has been approved by FDA and EMA in 2022 for treatment of patients with metastatic castration-resistant PCa (Sartor et al. 2021). In the present study, we demonstrate that the precursor PSMA-617 used for the synthesis of ^177^Lu-PSMA-617 inhibits cell proliferation and cell growth of LNCaP cells via its binding to PSMA. The mechanisms of the anti-proliferative effects include the down regulation of cyclin D1 and cyclin E1 and the increase of the cyclin-dependent kinase inhibitor, p21, resulting in the cell cycle arrest in the G1 stage. Although PSMA-617 alone does not induce death of tumour cells in our experimental setting, it significantly potentiates the cell death-promoting effect of ^177^Lu-PSMA-617 in LNCaP cells at concentrations which do not reduce the binding of ^177^Lu-PSMA-617.

### Anti-proliferative effects of PSMA-617 in LNCaP cells

Increased expression rates and clustering of PSMA in PCa aberrantly activate the phosphatidylinositol 3-kinase (PI3K)/AKT pathway instead of the canonical mitogen-activated protein kinase (MAPK) pathway. Along with an over-expression of growth factor receptors, like the insulin-like growth factor receptor, the over-activation of the PI3/AKT pathway results in cell cycle progression by regulating of the cyclin gene expression and the activity of CDKs (Perico et al. 2016; Caromile and Shapiro 2017; Wang 2021). Besides the role of cyclin D1 in the DNA repairing processes in some cell types, this cyclin unequivocally supports cell proliferation of prostate cancer cells (Ju et al. 2014; Reutens et al. 2001). Cyclin E1 promotes tumourigenesis in various cell types (for review see Hwang and Clurman 2005). Both cyclins play a critical role in the G1/S transition. After binding of cyclin D1 and cyclin E to the corresponding kinases, CDK4 and CDK2, respectively, and the formation of the maturation-promoting factor, they, together with several transcription factors, enable the expression of genes that regulate the entry of cells into the S phase (Malumbres and Barbacid 2001). The down-regulation of cyclin D1 and cyclin E1 by PSMA-617 prevents the formation and activation of the corresponding cyclin/CDK complexes and the maturation-promoting factors, which in turn arrests the G1/S transition. We have shown that PSMA-617 impaired the DNA duplication and, consequently, cell proliferation.

### p21 and p27 and the cell cycle in LNCaP cells

The CDK inhibitors, p21 and p27, bind to cyclin-CDK complexes to inhibit their catalytic activity. p21 inhibits cyclin-CDK2, cyclin-CDK1 and cyclin-CDK4,6 complexes and thus inhibits the cell cycle progression during G1 and S phases. The prognostic significance of the rate of p21 expression in PCa is controversially discussed. A p21 overexpression was associated with poor clinical outcome (Baretton et al. 1999). On the other hand, an up-regulation of p21 was demonstrated to induce growth arrest in several PCa cell lines (Roy et al. 2009; Lee et al. 2008). p21 also inhibits the DNA replication via binding to the proliferating cell nuclear antigen (PCNA) (Abbas and Dutta 2009). We report that PSMA-617 up-regulates p21. Consequently, a number of tumour cells entered the quiescent G0 state and did not continue to proliferate, an effect also observed in the present study. The increase in p21 may also be intrinsically related to the reduced levels of cyclin E1, as the E3 ubiquitin ligase complexes, SCF^SKP2^, CRL4^CDT2^ and APC/C^CDC20^, promote the ubiquitylation and degradation of p21 only when it is bound by complexes of CDK2 with cyclin E or PCNA (Abbas and Dutta 2009).

p27 binds and inhibits cyclin/CDK complexes and arrests cell cycle. Therefore, p27 is a putative tumour-suppressor and its reduced expression has been shown to correlate with poor prognosis in cancer patients (Razavipour et al. 2020; Sgambato et al. 2000). However, we did not observe any alterations in p27 levels after treatment of LNCaP cells with PSMA-617.

### Potentiation of the cell death—promoting effects of ^177^

In our study, exposure of LNCaP cells to PSMA-617 did not reduce the expression of PSMA, the target molecule for ^177^Lu-PSMA-617, nor did it alter the uptake of the radioligand into the cells. Competitive binding analyses of PSMA-617 conducted in LNCaP membranes by Tönnesmann et al. (2019) or Ruigrok et al. (2021) revealed that approximately 10 nM PSMA-617 concentration already reduced the ^177^Lu-PSMA-binding by 50%. In contrast, we demonstrate here that the unlabelled precursor up to 100 nM concentration does not affect the uptake of the ^177^Lu-PSMA-617 into cultured LNCaP cells. Different concentrations of ^177^Lu-PSMA-617 and different incubation times may account for the observed discrepancies. Furthermore, the binding of the ligand to cell membranes does not necessarily need to correlate with its uptake into living cells. Our results indicate that that PSMA-617 administered to patients at the clinically relevant doses does not interfere with the ^177^Lu-PSMA-617 endocytosis.

The most prominent finding of this study is the considerable potentiation of the cell death-inducing activity of ^177^Lu-PSMA-617 by PSMA-617. Upon inhibitor binding, PSMA internalises via clathrin—coated pits and subsequent endocytosis results in an effective transportation of the PSMA-inhibitor complex into the cell (Sheehan et al. 2022; Matthias et al. 2021; Ghosh and Heston 2004). After internalisation, the inhibitor is released and homogenously distributed in the cytoplasm, PSMA is recycled. The perinuclear enrichment of a radionuclide labelled inhibitor ensures an efficient radiation-mediated damage of PCa cells (Matthias et al. 2021; El Fakiri et al. 2021). Therefore, the endocytosis of the ^177^Lu-PSMA-617/PSMA- and PSMA-617/PSMA complexes is the prerequisite for their anti-cancer effects. After binding and intracellular internalisation of ^177^Lu-PSMA-617, tumour cells are destroyed by ^177^Lu through the delivery of the beta particle radiation. The underlying mechanisms involve an effective alteration of the DNA structure, e.g. single strange DNA-damage and/or related molecular signalling pathways related to DNA repairing processes (reviewed by El Fakiri et al. 2021). In addition, PSMA-617 promotes cell-cycle arrest (see above). Both, the anti-proliferative activity of PSMA-617 and the cell death-promoting effects of ^177^Lu-PSMA-617 result in a substantial elimination of tumour cells.

Interestingly, ^177^Lu-PSMA-617 at a dose of 200 KBq/ml alone, which is the lowest dose inducing cell death in LNCaP cell, did not exert any cytotoxic effects after a 48 h exposure to the radioligand. We do not have any plausible explanation for this rather surprising finding. We assume that the majority of the radiosensitive cells were already killed during the 24 h exposure to the radioligand. The remaining cells most probably entered the stage in which the tumour cells exhibit marked radioresistance. Nevertheless, the simultaneous treatment of LNCaP cells with the radioligand and its precursor, PSMA-617, did induce cell death. This finding substantiates the relevance of the PSMA-617 for the cytotoxic effects of ^177^Lu-PSMA-617 in PCa cells.

## Conclusion

Our results indicate that PSMA-617 inhibits proliferation of PCa cells and potentiates the cell death-promoting effects of ^177^Lu-PSMA-617. These findings require further confirmation in vitro and in vivo experiments to study the exact molecular mechanisms associated with the growth-inhibitory effects of PSMA-617 observed in PCa cells in order to test the potential of the simultaneous treatment with PSMA-617 and ^177^Lu-PSMA-617 for the translation in a clinical trial.

## Data Availability

The datasets can be accessed from Dr. Yi Zhao.
